# Intrauterine Infection and Preterm Labor

**DOI:** 10.1155/S1064744995000287

**Published:** 1995

**Authors:** Manju Monga, Jorge D. Blanco

**Affiliations:** Department of Obstetrics, Gynecology, and Reproductive Sciences University of Texas Medical School at Houston 6431 Fannin Suite 3.204 Houston TX 77030 USA

## Abstract

Preterm birth remains the leading cause of perinatal mortality and morbidity. Evidence suggests that intrauterine infection plays an important role in the pathogenesis of preterm labor. This article reviews the clinical data supporting this theory and the cellular and biochemical mechanisms by which intrauterine infection may initiate uterine contractions. The clinical and laboratory methods of diagnosing clinical chorioamnionitis and asymptomatic bacterial invasion of the intraamniotic cavity are also reviewed. Finally, the management of clinical chorioamnionitis and asymptomatic microbial invasion of the amniotic fluid and the use of adjunctive antibiotic therapy in the treatment of preterm labor are presented.


Infectious Diseases in Obstetrics and Gynecology 3:37–44 (1995)
© 1995 Wiley-Liss, Inc.

### Intrauterine Infection and Preterm Labor

**Manju Monga and Jorge D. Blanco**

*Department of Obstetrics, Gynecology, and Reproductive Sciences, University of Texas Medical School at Houston, Houston, TX*

ABSTRACT

**Preterm birth remains the leading cause of perinatal mortality and morbidity. Evidence suggests that intrauterine infection plays an important role in the pathogenesis of preterm labor. This article reviews the clinical data supporting this theory and the cellular and biochemical mechanisms by which intrauterine infection may initiate uterine contractions. The clinical and laboratory methods of diagnosing clinical chorioamnionitis and asymptomatic bacterial invasion of the intraamniotic cavity are also reviewed. Finally, the management of clinical chorioamnionitis and asymptomatic microbial invasion of the amniotic fluid and the use of adjunctive antibiotic therapy in the treatment of preterm labor are presented.** © 1995 Wiley-Liss, Inc.

KEY WORDS

Chorioamnionitis, microbial invasion of the amniotic cavity, pregnancy

Preterm birth remains the leading cause of perinatal mortality and morbidity. Eighty-three percent of the neonatal mortality in nonanomalous infants occurs in infants born before the 37th week of gestation.[1] Premature infants who survive the perinatal period experience significant morbidity including respiratory distress, intraventricular hemorrhage, sepsis, necrotizing enterocolitis, and patent ductus arteriosus.[2] Despite the well-recognized risks of preterm birth and concerted efforts to successfully arrest preterm labor, the incidence of preterm delivery has not decreased over the past decade, remaining at 9.6%.[3]

Recent research focusing on the pathogenesis of preterm labor suggests that intrauterine infection may play an important role in preterm labor and delivery. This article reviews the clinical, cellular, and biochemical data that support this theory, as well as the clinical and laboratory methods of diagnosing clinical chorioamnionitis and asymptomatic bacterial invasion of the intraamniotic cavity. Finally, the management issues regarding the treatment and role of adjunctive antibiotic therapy in preterm labor are presented.

The term "intrauterine infection" includes both clinical and asymptomatic microbial invasion of the amniotic cavity. For the purpose of this review "histologic chorioamnionitis" is defined as histologic inflammation of the amnion and chorion and "clinical chorioamnionitis" is reserved for the clinical syndrome associated with microbial invasion of the intraamniotic cavity.

### INTRAUTERINE INFECTION AND PRETERM DELIVERY

### Clinical Evidence

A review of 15 studies in which amniocenteses were performed on women in preterm labor with intact membranes reveals the rate of microbial invasion of the amniotic cavity to be 12.2%.[4–18] The bacterial isolation rates range from 0 to 48%, depending on the study population and culture technique. Although most of these women had no signs or symptoms of infection at the time of presentation, they

Address correspondence/reprint requests to Dr. Manju Monga, Department of Obstetrics, Gynecology, and Reproductive Sciences, University of Texas Medical School at Houston, 6431 Fannin, Suite 3.204, Houston, TX 77030.

Received June 20, 1994
Accepted October 19, 1994

Review Article were more likely to develop clinical chorioamnionitis. Furthermore, women in preterm labor with positive cultures were more likely to be refractory to tocolytics and to have spontaneous premature rupture of the membranes (PROM) and a shorter interval from presentation to delivery. This evidence indirectly supports the role of infection in the pathogenesis of preterm labor with intact membranes. Alternatively, these data could be interpreted to suggest that women with preterm labor are at increased risk for microbial invasion of the amniotic cavity.

PROM accounts for up to one-third of all preterm deliveries. Seven studies have examined the rate of intrauterine infection by performing transabdominal amniocentesis on women with PROM.[19–25] Transabdominal amniocenteses were successfully performed on 59% of these women, 28% of whom had positive amniotic fluid cultures. The rate of microbial invasion of the amniotic cavity in the women with unsuccessful amniocenteses is unknown but theoretically higher than 28%, as women with PROM and oligohydramnios are more likely to develop chorioamnionitis[23,26] but less likely to have a successful amniocentesis due to technical difficulty.[19–25] Therefore, these studies support the association of intrauterine infection and PROM, although a causal relationship has not been clearly established. One study documented a higher incidence of positive cultures in women who presented with PROM and preterm labor (39%) compared with women with PROM alone (25%).[25] Eighty-one of 160 women with negative cultures who were not in labor at the time of admission subsequently developed preterm labor. Of these 81 women, 48 had repeat amniocenteses at the onset of labor, and 75% had positive cultures at that time.[25] This suggests that intrauterine infection may play a pivotal role in the onset of preterm labor associated with PROM.

Preterm delivery is associated with an increased rate of postpartum endometritis and early neonatal sepsis,[27–29] which provides further circumstantial evidence to support intrauterine infection as an etiological factor in preterm labor and delivery.

The pathologic examination of the placenta and membranes revealed an increased rate of histologic chorioamnionitis following premature delivery (up to 60%) compared with the rate following term delivery (up to 20%).[30,31] As histologic chorioamnionitis is correlated with positive amniotic-fluid cultures, the hypothesis is that intrauterine infection, with resulting inflammation of the placental membranes, stimulates the onset of preterm labor.

The pathogenesis of intrauterine infection appears to be related to ascending pathogens. An association between genitourinary colonization with group B streptococcus[32,33] and *Neisserisa gonorrhoeae*[34–36] and premature delivery has been established. In addition, the eradication of these pathogens with antibiotics has significantly decreased the preterm delivery rate.[37,38] Bacterial vaginosis and cervicovaginal colonization with *Bacteroides fragilis* have also been associated with preterm delivery, although their eradication has yet to demonstrate a significant benefit.[31,39–44] The role of cervicovaginal *Chlamydia trachomatis*, *Ureaplasma urealyticum*, and *Trichomonas vaginalis* on prematurity is less well established.

### Biochemical Mechanisms

Prostaglandins have been implicated in the initiation of labor at term. This theory is supported by the observations that 1) prostaglandins and their precursor, arachidonic acid, stimulate abortion and labor;[45] 2) amniotic-fluid levels of prostaglandins and arachidonic acid increase during labor;[46–49] and 3) prostaglandin inhibitors arrest preterm contractions and delay the onset of labor at term.[50–52] The role of prostaglandins in the onset of preterm labor is less well defined. However, women with preterm labor and microbial invasion of the amniotic cavity have higher amniotic fluid levels of $PGE_2$ and $PGF_{2\alpha}$ than women with preterm labor and negative amniotic-fluid cultures or women without preterm labor.[53–58] The sources of these prostaglandins are postulated to be the amnion, chorion, and decidua.[59–61] Indeed, media from bacterial cultures stimulate $PGE_2$ and $PGF_{2\alpha}$ production from isolated human amnion and decidual cells.[62–65] Therefore, prostaglandins appear to play an important role in preterm labor in the presence of infection.

Prostaglandin production from placental membranes and decidual cells increases directly in response to bacterial products such as phospholipase $A_2$ and phospholipase C. These stimulate the release of arachidonic acid from the membrane phospholipids, phosphatidylinositol and phosphatidylethanolamine.[66–68] Endotoxin, or lipopolysaccharide, a component of the cell wall of gram-negative bacteria, stimulates prostaglandin production by the amnion and decidua[69,70] and is increased in the amniotic fluid of women with microbial invasion of the amniotic cavity[71] or PROM and labor.[72] However, the amount of endotoxin found in the amniotic cavity in women with PROM and labor[72] is lower than that required to stimulate prostaglandin production by the amnion.[69] Therefore, it has been postulated that an amplification of the host response by endotoxin stimulates the production of inflammatory mediators.

Endotoxin stimulates the production of inflammatory mediators, cytokines or monokines, from macrophages.[73–76] The 3 cytokines that have been extensively investigated in relation to preterm labor and intrauterine infection are interleukin-1, interleukin-6, and tumor-necrosis factor. These cytokines stimulate the production of prostaglandins by the amnion, chorion, decidua, and myometrium in vitro.[73,75,77–81] The amniotic-fluid levels of interleukin-1, interleukin-6, and tumor-necrosis factor are higher in women with microbial invasion of the amniotic cavity and preterm labor compared with the levels in women with microbial invasion of the amniotic cavity who are not in labor or women without microbial invasion of the amniotic cavity.[73,75,77–81] Elevated cytokine levels are also associated with increased amniotic-fluid levels of prostaglandins and unsuccessful tocolysis.[75,82–84] Finally, the systemic administration of interleukin-1 in mice induces preterm delivery,[85] which can be inhibited by the administration of a natural interleukin-1 receptor antagonist.[86] The amniotic-fluid levels of interleukin-1 receptor antagonist are reduced in women with intraamniotic-fluid infection and preterm labor despite elevated levels of interleukin-1.[87]

The sum of these data suggests that microbial invasion of the amniotic cavity results in a release of bacterial endotoxin which stimulates cytokine production and results in arachidonic-acid metabolism, prostaglandin production, and the onset of labor. However, the simultaneous or sequential production of several cytokines may be necessary to stimulate this cascade through a positive feedback mechanism[88,89] or a prolonged exposure to elevated levels of cytokines may be necessary, as individual cytokines do not acutely stimulate myometrial contractile activity in vitro.[90]

### DIAGNOSIS OF INTRAUTERINE INFECTION

A diagnosis of clinical chorioamnionitis is based upon fever >37.8°C; rupture of membranes; and 2 or more of the following criteria: maternal or fetal tachycardia, uterine tenderness, malodorous amniotic fluid, or peripheral leukocytosis with no other site of infection.[91,92]

The gold standard for the diagnosis of subclinical intrauterine infection has been the amniotic-fluid culture. Because the results are usually not obtainable for 48–72 h, several techniques for the rapid identification of microbial invasion of the amniotic cavity have been investigated.

A Gram's stain of the amniotic fluid is a simple and inexpensive test to perform. Numerous reports have shown it to have a sensitivity of about 49.5% (140/283 patients) and a specificity of 97.7% (888/909 patients) when compared with the amniotic-fluid culture in the setting of preterm labor with intact membranes or PROM.[4,5,8,11,13,16,19–22,24,93–97] The low sensitivity of the Gram's stain to detect microbial invasion of the amniotic cavity is probably related to its inability to demonstrate mycoplasmas which are among the most common isolates in asymptomatic microbial invasion of the amniotic cavity.[97]

Low amniotic-fluid glucose levels have been investigated as a rapid, simple, and inexpensive indicator of microbial invasion of the amniotic cavity. With a cutoff level of <14–16 mg/dl, this technique has been found to have a sensitivity of 33–92% and a specificity of 58–94% in women with preterm labor or PROM when compared with amniotic-fluid cultures.[94–98] Due to the wide variation in sensitivity and specificity, the clinical utility of amniotic-fluid glucose levels in identifying microbial invasion of the amniotic cavity is low.

An amniotic-fluid white blood cell (WBC) count of ≥50 cells/mm$^3$ has been shown to have a sensitivity of 64% and specificity of 94% when compared with amniotic-fluid culture for identifying microbial invasion of the amniotic cavity in women with preterm labor and intact membranes.[97] The amniotic-fluid WBC count may be useful in women with asymptomatic microbial invasion of the amniotic fluid, but otherwise has little clinical value. The leukocyte esterase assay of amniotic fluid (a measure of leukocyte activity) was shown to have a higher sensitivity (81%) in one study,[99] but this report has not been confirmed.[95]

A laboratory technique, currently unavailable for clinical use in most centers, is the determination of amniotic-fluid interleukin-6 levels. Elevated amniotic-fluid interleukin-6 levels have been found to be more predictive of microbial invasion of the amniotic cavity, amniocentesis-to-delivery interval, and neonatal complications than the Gram's stain, glucose concentration, or WBC count in women with preterm labor and intact membranes and in women with PROM.[96,97,100] However, there are no studies documenting a benefit from intervention in the setting of preterm labor or PROM with elevated cytokine levels. Therefore, the clinical relevance of these reports is uncertain.

### MANAGEMENT

### Clinical Chorioamnionitis

Once the diagnosis of clinical chorioamnionitis has been made, antibiotic therapy should be instituted. Prompt intrapartum treatment clearly decreases the rate of neonatal and maternal morbidity.[101] As chorioamnionitis is a polymicrobial infection, the combination therapy of ampicillin and gentamicin is effective.[101–103] Single-agent therapy using second- or third-generation cephalosporins or extended-spectrum penicillins has not been studied in large randomized trials. A critical diagnosis-to-delivery interval has not been determined; however, there are little data on the outcome in patients who deliver >12 h after diagnosis. It has been recommended that clindamycin be added to the antibiotic regimen if the patient is delivered by cesarean in an attempt to decrease the severity of postpartum endometritis, although little data exist to support this recommendation.[91]

### Asymptomatic Microbial Invasion of the Amniotic Fluid

While the early identification of microbial invasion of the amniotic cavity raises the possibility of many innovative therapeutic options, such as antibiotics without prompt delivery or anticytokines, these options have not been investigated. Many patients with preterm labor and microbial invasion of the amniotic cavity are refractory to tocolysis, and women with PROM and microbial invasion of the amniotic cavity have shorter latency periods. The management of a patient with preterm labor or PROM who has a positive amniotic-fluid culture without clinical chorioamnionitis has not been addressed in the literature. In our clinical practice, we do not routinely perform an amniocentesis for an amniotic-fluid culture in a woman with preterm labor or a woman with PROM. However, we recommend an amniotic-fluid culture in the case of refractory preterm labor. In this situation, we initiate antibiotic therapy and proceed toward delivery if the amniotic-fluid culture is positive. Due to the paucity of data in this area, these management decisions should be individualized.

### Adjunctive Antibiotic Therapy in Preterm Labor

The large body of evidence that supports an etiologic role of infection in preterm labor has prompted several investigations of adjuvant antibiotics for women with preterm labor. To date, the reported results have been contradictory. McGregor et al.[104] randomized 17 patients to a β-sympathomimetic agent plus placebo or β-sympathomimetic agent plus erythromycin and found a statistically significant increase in the number of women who delivered at term in the erythromycin-treated group. Morales et al.[105] randomized 150 women with "idiopathic preterm labor" to ampicillin, erythromycin, or no treatment. They found a statistically significant prolongation of pregnancy in the antibiotic-treated groups; however, the administration of a placebo was not included in the study design, a wide range was noted in the number of days of prolongation of gestation, and no significant improvement was shown in the proportion of pregnancies reaching 37 weeks gestation. McGregor et al.[106] reported a prolongation of pregnancy (35 vs. 25 days) in 103 women receiving tocolytics for preterm labor who were randomized to receive clindamycin or placebo.

In contrast to these 3 studies, Newton et al.[107] found no difference in the prolongation of pregnancy or outcome in 78 women randomized to receive either ampicillin plus erythromycin or placebo in conjunction with standard tocolytics. They reported similar results in 86 patients receiving magnesium-sulfate tocolysis who were randomized to receive ampicillin-sulbactam plus indomethacin or placebo.[108] Recently, in a multicenter double-blind, placebo-controlled trial, 277 women on tocolytic therapy for preterm labor were randomized to receive either ampicillin plus erythromycin or placebo.[109] No difference was found in the rate of preterm delivery, clinical chorioamnionitis, endometritis, or neonatal outcome. In this study, only 5.8% of the 239 women who had amniocenteses had positive amniotic-fluid cultures.

Basic and clinical evidence supports the etiologic role of infection in some cases of preterm labor. Intrauterine infection is often present in women with preterm labor or PROM, and bacterial products such as endotoxin and cytokines stimulate prostaglandin production. These data suggest a role for antibiotics in the prevention of preterm birth. As preterm labor is probably multifactorial,[110] it is unlikely that adjunctive antibiotic therapy will be of any therapeutic benefit in those women in whom infection is not present. Clearly, there is a need for a large randomized placebo-controlled study on the effect of adjunctive antibiotic therapy in women in preterm labor and intact membranes with asymptomatic microbial invasion of the amniotic cavity.

### ACKNOWLEDGMENTS

M.M. is supported in part by the Burroughs Wellcome Fellowship of the American Gynecological and Obstetrical Foundation.

### REFERENCES

1. Cooper RL, Goldenberg RL, Creasy RK, et al.: A multicenter study of preterm birth weight and gestational age specific mortality. Am J Obstet Gynecol 168: 78–84, 1993.
2. Robertson PA, Sniderman SH, Laros RK, et al.: Neonatal morbidity according to gestational age and birth weight from five tertiary centers in the United States, 1983 through 1986. Am J Obstet Gynecol 166:1629–1645, 1992.
3. Advance Report on final natality statistics, 1989. Monthly vital statistics report. 40 (suppl):1–53, 1991.
4. Miller JM, Pupkin MJ, Hill GB: Bacterial colonization of amniotic fluid from intact fetal membranes. Am J Obstet Gynecol 136:796–804, 1980.
5. Bobitt JR, Hayslip CC, Damato JD: Amniotic fluid infection as determined by transabdominal amniocentesis in patients with intact membranes in premature labor. Am J Obstet Gynecol 140:947–952, 1981.
6. Wallace RL, Herrick CN: Amniocentesis in the evaluation of premature labor. Obstet Gynecol 57:483–486, 1981.
7. Wahbeh CJ, Hill GB, Eden RD, Gall SA: Intra-amniotic infection in asymptomatic patients with refractory preterm labor. Obstet Gynecol 148:739–743, 1984.
8. Hameed C, Tejani N, Verma UL, Archbald F: Silent chorioamnionitis as a cause of preterm labor refractory to tocolytic therapy. Obstet Gynecol 149:726–730, 1984.
9. Weibel DR, Randall HW: Evaluation of amniotic fluid in preterm labor with intact membranes. J Reprod Med 30:777–780, 1985.
10. Gravett MG, Hummel D, Eschenbach DA, Holmes KK: Preterm labor associated with subclinical amniotic fluid infection and with subclinical bacterial vaginosis. Obstet Gynecol 67:229–237, 1986.
11. Duff P, Kopelman JN: Subclinical intra-amniotic infection in asymptomatic patients with refractory preterm labor. Obstet Gynecol 69:756–759, 1987.
12. Iams JD, Clapp DH, Contos DA, Whitehurst R, Ayers LW, O'Shaughnessy RW: Does extra-amniotic infection cause preterm labor? Gas-liquid chromatography studies of amniotic fluid in amnionitis, preterm labor and normal controls. Obstet Gynecol 70:365–368, 1987.
13. Romero R, Emamian M, Quintero R, et al.: The value and limitations of the gram stain examination in the diagnosis of intraamniotic infection. Am J Obstet Gynecol 159:114–119, 1988.
14. Skoll MA, Moretti ML, Sibai BM: The incidence of positive amniotic fluid cultures in patients in preterm labor with intact membranes. Am J Obstet Gynecol 161:813–816, 1989.
15. Leigh J, Garite TJ: Amniocentesis and the management of premature labor. Obstet Gynecol 67:500–506, 1989.
16. Romero R, Sirtori M, Oyarzun E, et al.: Infection and labor. V. Prevalence, microbiology, and clinical significance of intraamniotic infection in women with preterm labor and intact membranes. Am J Obstet Gynecol 161: 817–824, 1989.
17. Harger JH, Meyer MP, Amortegui A, MacPherson TA, Kaplan L, Mueller-Heubach E: Low incidence of positive amniotic fluid cultures in preterm labor at 27–32 weeks in the absence of clinical evidence of chorioamnionitis. Obstet Gynecol 77:228–234, 1991.
18. Watts D, Krohn MA, Hillier SL, Eschenbach DA: The association of occult amniotic fluid infection with gestational age and neonatal outcome among women in preterm labor. Obstet Gynecol 79:351–357, 1992.
19. Garite TJ, Freeman RK, Linzey EM, Braly P: The use of amniocentesis in patients with premature rupture of membranes. Obstet Gynecol 54:226–230, 1979.
20. Garite TJ, Freeman RK: Chorioamnionitis in the preterm gestation. Obstet Gynecol 59:539–545, 1982.
21. Cotton CB, Hill LM, Strassner HT, Platt LD, Ledger WJ: Use of amniocentesis in preterm gestation with ruptured membranes. Obstet Gynecol 63:38–43, 1984.
22. Broekhuizen FF, Gilman M, Hamilton PR: Amniocentesis for gram stain and culture in preterm premature rupture of the membranes. Obstet Gynecol 66:316–321, 1985.

23. Vintzileos AM, Campbell WA, Nochimson DJ, Weinbaum PJ, Escoto DT, Mirochnick MH: Qualitative amniotic fluid volume versus amniocentesis in predicting infection in preterm rupture of the membranes. Obstet Gynecol 67:579–583, 1986.
24. Feinstein ST, Vintzileos AM, Lodeiro JG, Campbell WA, Weinbaum PJ, Nochimson DJ: Amniocentesis with premature rupture of membranes. Am J Obstet Gynecol 68:147–152, 1986.
25. Romero R, Quintero R, Oyarzun E, et al.: Intraamniotic infection and the onset of labor in preterm premature rupture of membranes. Am J Obstet Gynecol 159: 661–666, 1988.
26. Gonik B, Bottoms SF, Cotton DB: Amniotic fluid volume as a risk factor in preterm premature rupture of the membranes. Obstet Gynecol 65:456–459, 1985.
27. Daikoku NH, Kaltreider DF, Khouzami VA, Spence M, Johnson JWC: Premature rupture of membranes and spontaneous preterm labor: Maternal endometritis risks. Obstet Gynecol 59:13–20, 1982.
28. McCracken G, Shinefield H: Changes in the pattern of neonatal septicemia and meningitis. Am J Dis Child 112:33–39, 1966.
29. Seo K, McGregor JA, French JI: Preterm birth is associated with increased risk of maternal and neonatal infection. Obstet Gynecol 79:75–80, 1992.
30. Guzick DS, Winn K: The association of chorioamnionitis with preterm delivery. Obstet Gynecol 65:11–16, 1985.
31. Hillier SL, Martius J, Krohn M, Kiviat N, Holmes KK, Eschenbach DA: A case-control study of chorioamnionic infection and histologic chorioamnionitis in prematurity. N Engl J Med 31:972–978, 1988.
32. Moller M, Thomsen AC, Borch K, et al.: Premature delivery and group B streptococcal bacteriuria. Lancet 2:69–71, 1984.
33. White CP, Wilkins EGL, Roberts C, et al.: Premature delivery and group B streptococcal bacteriuria. Lancet 2:586, 1984.
34. Handsfield HH, Hodson A, Holmes KK: Neonatal gonococcal infection: Orogastric contamination with *Neisseria gonorrhoeae*. JAMA 225:697–701, 1973.
35. Amstey MS, Steadman KT: Symptomatic gonorrhea and pregnancy. J Am Vener Dis Assoc 3:14–16, 1976.
36. Edwards LE, Barrada MI, Hammann AA, Hankanson EY: Gonorrhea in pregnancy. Am J Obstet Gynecol 132:637–642, 1978.
37. Thomsen AC, Morup L, Hansen KB: Antibiotic elimination of group B streptococci in urine in prevention of preterm labour. Lancet 1:591–596, 1987.
38. Elliott B, Brunham RC, Laga M, et al.: Maternal gonococcal infection as a preventable risk factor for low birth weight. J Infect Dis 161:531–536, 1990.
39. Minkoff H, Grunebaum AN, Schwarz RH, et al.: Risk factors for prematurity and premature rupture of membranes: A prospective study of the vaginal flora in pregnancy. Am J Obstet Gynecol 150:965–972, 1984.
40. Martius J, Krohn MA, Hillier SL, et al.: Relationships of vaginal *Lactobacillus* species, cervical *Chlamydia trachomatis,* and bacterial vaginosis to preterm birth. Obstet Gynecol 71:89–92, 1988.
41. McDonald HM, O'Loughlin JA, Jolley P, Vigneswaran P, McDonald PH: Vaginal infections and preterm labour. Br J Obstet Gynaecol 98:427–435, 1991.
42. Krohn MA, Hillier SL, Lee ML, Rabe LK, Eschenbach DA: Vaginal *Bacteroides* are associated with an increased rate of preterm delivery among women in preterm labor. J Infect Dis 164:88–93, 1991.
43. Gravett MG, Nelson HP, DeRouen T, et al.: Independent associations of bacterial vaginosis and *Chlamydia trachomatis* infection with adverse pregnancy outcome. JAMA 256:1899–1903, 1986.
44. McGregor JA, French JI, Richter R, et al.: Antenatal microbiologic and maternal risk factors associated with prematurity. Am J Obstet Gynecol 163:1465–1473, 1990.
45. MacDonald PC, Schultz FM, Duenhoelter JH, et al.: Initiation of human parturition. Obstet Gynecol 44:629–641, 1979.
46. Karim SMM, Devlin J: Prostaglandin content of amniotic fluid during pregnancy and labour. J Obstet Gynaecol Br Commonw 74:230–234, 1967.
47. Keirse MJ, Flint AP, Turnbull AC: F prostaglandins in amniotic fluid during pregnancy and labour. J Obstet Gynaecol Br Commonw 81:131–135, 1974.
48. Dray F, Frydman R: Primary prostaglandins in amniotic fluid in pregnancy and spontaneous labor. Am J Obstet Gynecol 125:13–17, 1976.
49. Keirse MJNC, Hicks BR, Mitchell MD, Turnbull AC: Increase of the prostaglandin precursor, arachidonic acid, in amniotic fluid during spontaneous labour. Br J Obstet Gynaecol 84:937–940, 1977.
50. Zuckerman H, Reiss U, Rubinstein I: Inhibition of human premature labor by indomethacin. Obstet Gynecol 44:787–794, 1974.
51. Lewis RB, Schulman JD: Influence of acetylsalicylic acid, an inhibitor of prostaglandin synthesis on the duration of human gestation and labour. Lancet 2:1159–1161, 1973.
52. Novy MJ, Cook MJ, Manaugh L: Indomethacin block of normal onset of parturition in primates. Am J Obstet Gynecol 118:412–415, 1974.
53. TambyRaja RL, Salmon JA, Karim SM, Ratnam SS: F prostaglandin levels in amniotic fluid in premature labor. Prostaglandin 13:339–348, 1977.
54. Weitz CM, Ghodgaonkar RB, Dubin NH, Niebyl JR: Prostaglandin F metabolite concentration as a prognostic factor in preterm labor. Obstet Gynecol 67:496–500, 1986.
55. Romero R, Quintero R, Emamian M, Wan M, Hobbins JC, Mitchell MD: Prostaglandin concentrations in amniotic fluid of women with intraamniotic infection and preterm labor. Am J Obstet Gynecol 157:1461–1467, 1987.

56. Romero R, Wu YK, Mazor M, Hobbin JC, Mitchell MD: Amniotic fluid prostaglandin $E_2$ in preterm labor. Prostaglandins Leuk Essen Fatty Acids 34:141–145, 1988.
57. Romero R, Wu YK, Sirtori M, et al.: Amniotic fluid concentrations of prostaglandins F2 alpha, 13,14-dihydro-15-keto-11, 16-cyclo prostaglandin E2 (PGEM-ii) in preterm labor. Prostaglandins 37:149–161, 1989.
58. Lopez-Bernal A, Hansell DJ, Khong TY, Kheeling JW, Turnbull AC: Prostaglandin E production by the fetal membranes in unexplained preterm labor and preterm labor associated with chorioamnionitis. Br J Obstet Gynaecol 96:1133–1139, 1989.
59. Kinoshita K, Satoh K, Sakamoto S: Biosynthesis of prostaglandin in human decidua, amnion, chorion, and villi. Endocrinol Jpn 24:343–340, 1977.
60. Willman EA, Collins WP: The metabolism of prostaglandin $E_2$ by tissues from the human uterus and foetoplacental unit. Acta Endocrinol 87:632–642, 1978.
61. Mitchell MD, Bibby JG, Hicks BR, Turnbull AC: Specific production of prostaglandin E by human amnion in vitro. Prostaglandins 15:377–380, 1978.
62. Lamont RF, Rose M, Elder MG: Effects of bacterial products on prostaglandin E production by amnion cells. Lancet 2:1131–1133, 1985.
63. Bennett PR, Rose MP, Myatt L: Preterm labor: Stimulation of arachidonic acid metabolism in human amnion by bacterial products. Am J Obstet Gynecol 156:649–655, 1987.
64. Lamont RF, Anthony F, Myatt L, Booth L, Furr PM, Taylor-Robinson D: Production of prostaglandin $E_2$ by human amnion in vitro in response to addition of media conditioned by microorganisms associated with chorioamnionitis and preterm labor. Am J Obstet Gynecol 162:819–825, 1990.
65. Mitchell MD, Romero RJ, Avila C, Foster JT, Edwin SS: Prostaglandin production by amnion and decidual cells in response to bacterial products. Prostaglandins Leuk Essen Fatty Acids 42:167–169, 1991.
66. Bejar R, Curbelo V, Davis C, Gluck L: Premature labor: Bacterial source of phospholipase. Obstet Gynecol 57:479–482, 1981.
67. Takahashi K, Imai A, Tamaya T: Preterm labor and bacterial intra-amniotic infection: Arachidonic acid liberation by the action of phospholipase $A_2$. Arch Gynecol Obstet 244:1–6, 1988.
68. McGregor JA, Lawellin D, Franco-Buff A, Todd JK: Phospholipase C activity in microorganisms associated with reproductive tract infection. Am J Obstet Gynecol 164:682–686, 1991.
69. Romero R, Hobbins JC, Mitchell MD: Endotoxin stimulates prostaglandin $E_2$ production by human amnion. Obstet Gynecol 71:227–228, 1988.
70. Romero R, Mazor M, Wu YK, Oyarzun AE, Mitchell MD: Bacterial endotoxin and tumor necrosis factor stimulate prostaglandin production by human decidua. Prostaglandins Leuk Essen Fatty Acids 37:183–185, 1989.
71. Romero R, Kadar N, Hobbins JC, et al.: Infection and labor: The detection of endotoxin in amniotic fluid. Am J Obstet Gynecol 157:815–819, 1987.
72. Romero R, Roslansky P, Oyarzun E, et al.: Labor and infection. II. Bacterial endotoxin in amniotic fluid and its relationship to the onset of preterm labor. Am J Obstet Gynecol 158:1044–1049, 1988.
73. Casey ML, Cox SM, Beutle B, Milewich L, MacDonald PC: Cachectin/tumor necrosis factor formation in human decidua: Potential role of cytokines in infection-induced preterm labor. J Clin Invest 83:430–436, 1989.
74. Romero R, Wu YK, Brody DT, Oyarzun E, Duff GW, Durum SK: Human decidua: A source of interleukin-1. Obstet Gynecol 73:31–34, 1989.
75. Romero R, Avila C, Santhanam U, Sehgal PB: Amniotic fluid interleukin 6 in preterm labor: Association with infection. J Clin Invest 85:1392–1400, 1990.
76. Mitchell MD, Edwin S, Romero R: Prostaglandin biosynthesis by human decidual cells: Effects of inflammatory mediators. Prostaglandins Leuk Essen Fatty Acids 41:35–38, 1990.
77. Romero R, Durum S, Dinarello CA, et al.: Interleukin-1 stimulates prostaglandin biosynthesis by human amnion. Prostaglandins 37:13–22, 1989.
78. Romero R, Manogue KR, Mitchell MD, et al.: Infection and labor. IV. Cachectin-tumor necrosis factor in the amniotic fluid of women with intraamniotic infection and preterm labor. Am J Obstet Gynecol 161:336–341, 1989.
79. Mitchell MD, Dudley DJ, Edwin SS, Schiller SL: Interleukin-6 stimulates prostaglandin production by human amnion and decidual cells. Eur J Pharmacol 192:189–191, 1991.
80. Lundin-Schiller S, Mitchell MD: Prostaglandin production by human chorion laeve cells in response to inflammatory mediators. Placenta 12:353–363, 1991.
81. Molnar M, Romero R, Hertelendy F: Interleukin-1, and tumor necrosis factor stimulate arachidonic acid release and phospholipid metabolism in human myometrial cells. Am J Obstet Gynecol 169:825–829, 1993.
82. Romero R, Brody DT, Oyarzun E, et al.: Infection and labor. III. Interleukin-1: A signal for the onset of parturition. Am J Obstet Gynecol 160:1117–1123, 1989.
83. Romero R, Mazor M, Brandt F, et al.: Interleukin-1$\alpha$ and interleukin-1$\beta$ in preterm and term human parturition. Am J Reprod Immunol 27:117–123, 1992.
84. Hillier SL, Witkin SS, Krohn MA, Watts DH, Kiviat NB, Eschenbach DA: The relationship of amniotic fluid cytokines and preterm delivery, amniotic fluid infection, histologic chorioamnionitis, and chorioamnion infection. Obstet Gynecol 81:941–948, 1993.
85. Romero R, Mazor M, Tartakovsky B: Systemic administration of interleukin-1 induces preterm parturition in mice. Am J Obstet Gynecol 165:969–971, 1991.
86. Romero R, Tartakovsky B: The natural interleukin-1 receptor antagonist prevents interleukin-1 induced preterm delivery in mice. Am J Obstet Gynecol 167:1041–1045, 1992.
87. Romero R, Sepulveda W, Mazor M, et al.: The natural interleukin-1 receptor antagonist in term and preterm parturition. Am J Obstet Gynecol 167:863–872, 1992.
88. Dudley DJ, Trautman MS, Araneo BA, Edwin SS,

Mitchell MD: Decidual cell biosynthesis of interleukin-6: Regulation by inflammatory cytokines. J Clin Endocrinol Metab 74:884–889, 1992.
89. Dudley DJ, Trautman MS, Edwin SS, Lundin-Schiller S, Mitchell MD: Biosynthesis of interleukin-6 by culture human chorion laeve cells: Regulation by cytokines. J Clin Endocrinol Metab 75:1081–1086, 1992.
90. Oshiro BT, Monga M, Eriksen NL, Graham JM, Weisbrodt NW, Blanco JD: Endotoxin, interleukin-1β, interleukin-6 or tumor necrosis factor-α do not acutely stimulate isolated murine myometrial contractile activity. Am J Obstet Gynecol 169:1424–1427, 1993.
91. Oshiro BT, Monga M, Blanco JD: Intraamniotic infections. Semin Perinatol 17:420–425, 1993.
92. Gibbs RS, Blanco JD, St. Clair PJ, Castaneda YS: Quantitative bacteriology of amniotic fluid from patients with clinical intraamniotic infection at term. J Infect Dis 145:1–8, 1982.
93. Zlatnik FJ, Cruikshank DP, Petzoid CR, Galask RP: Amniocentesis in the identification of inapparent infection in preterm patients with premature rupture of the membranes. J Reprod Med 29:656–669, 1984.
94. Gauthier DW, Meyer WJ, Bieniarz A: Correlation of amniotic fluid glucose concentration and intraamniotic infection in patients with preterm labor or premature rupture of membranes. Am J Obstet Gynecol 165:1105–1110, 1991.
95. Coultrip LL, Grossman JH: Evaluation of rapid diagnostic tests in the detection of microbial invasion of the amniotic cavity. Am J Obstet Gynecol 167:1231–1242, 1992.
96. Romero R, Yoon BH, Mazor M, et al.: A comparative study of the diagnostic performance of amniotic fluid glucose, white blood cell count, interleukin-6, and Gram stain in the detection of microbial invasion in patients with preterm premature rupture of membranes. Am J Obstet Gynecol 169:839–851, 1993.
97. Romero R, Yoon BH, Mazor M, et al.: The diagnostic and prognostic value of amniotic fluid white blood cell count, glucose, interleukin-6, and Gram stain in patients with preterm labor and intact membranes. Am J Obstet Gynecol 169:805–816, 1993.
98. Romero R, Jimenez C, Lohda AK, et al.: Amniotic fluid glucose concentration: A rapid and simple method for the detection of intraamniotic infection in preterm labor. Am J Obstet Gynecol 163:968–974, 1990.
99. Egley CC, Katz VL, Herbert WNP: Leukocyte esterase: A simple bedside test for the detection of bacterial colonization of amniotic fluid. Am J Obstet Gynecol 159:120–122, 1988.
100. Greig PC, Ernest JM, Teot L, Erikson M, Talley R: Amniotic fluid interleukin-6 levels correlate with histologic chorioamnionitis and amniotic fluid cultures in patients in premature labor with intact membranes. Am J Obstet Gynecol 69:1035–1044, 1993.
101. Gibbs RS, Dinsmoor MJ, Newton EER, Ramamurthy RS: A randomized trial of intrapartum versus immediate postpartum treatment of women with intra-amniotic infection. Obstet Gynecol 72:823–826, 1988.
102. Yoder RP, Gibbs RS, Blanco JD, et al.: A prospective controlled study of maternal and perinatal outcome after intra-amniotic infection at term. Am J Obstet Gynecol 145:695–701, 1983.
103. Gilstrap LC, Leveno KJ, Cox SM, Burris JS, Mashburn M, Rosenfeld CR: Intrapartum treatment of acute chorioamnionitis: Impact on neonatal sepsis. Am J Obstet Gynecol 159:579–583, 1988.
104. McGregor JA, French JI, Reller B, Todd JK, Makowski EL: Adjunctive erythromycin treatment for idiopathic preterm labor: Results of a randomized double-blinded, placebo-controlled trial. Am J Obstet Gynecol 154:98–103, 1986.
105. Morales WJ, Angel JL, O'Brien WF, Knuppel RA, Finazzo M: A randomized study of antibiotic therapy in idiopathic preterm labor. Obstet Gynecol 72:829–833, 1988.
106. McGregor JA, French JI, Seo K: Adjunctive clindamycin therapy for preterm labor: Results of a double-blind, placebo-controlled trial. Am J Obstet Gynecol 165:867–875, 1991.
107. Newton ER, Dinsmoor MJ, Gibbs RS: A randomized blinded, placebo-controlled trial of antibiotics in idiopathic preterm labor. Obstet Gynecol 74:562, 1989.
108. Newton ER, Shield L, Ridgway LE, Berkus MD, Elliott BD: Combination antibiotics and indomethacin in idiopathic preterm labor: A randomized double-blind clinical trial. Am J Obstet Gynecol 165:1753–1759, 1991.
109. Romero R, Sibai BM, Caritis S, et al.: Antibiotic treatment of preterm labor with intact membranes: A multicenter, randomized, double-blinded, placebo-controlled trial. Am J Obstet Gynecol 169:764–774, 1993.
110. Arias F, Rodrigues L, Rayne SC, et al.: Maternal placental vasculopathy and infection: Two distinct subgroups among patients with preterm labor and preterm ruptured membranes. Am J Obstet Gynecol 168:585–591, 1993.
